# ‘There is more to life than HbA_1c_ ’: A comprehensive qualitative framework from the Hypo‐RESOLVE project evidencing the impacts of hypoglycaemia on the quality of life of adults living with type 1 or type 2 diabetes

**DOI:** 10.1111/dme.70240

**Published:** 2026-02-09

**Authors:** Philip A. Powell, Sé M. Frances, Bastiaan E. de Galan, Simon Heller, Jane Speight, Myriam Rosilio, Frans Pouwer, Mari‐Anne Gall, Christopher J. Child, Rory J. McCrimmon, Ken Tait, Jill Carlton, the Hypo‐RESOLVE Consortium

**Affiliations:** ^1^ Sheffield Centre of Health and Related Research (SCHARR), University of Sheffield Sheffield UK; ^2^ Department of Medicine Radboud University Medical Centre Nijmegen GA The Netherlands; ^3^ Department of Internal Medicine Maastricht University Medical Center+ Maastricht ER The Netherlands; ^4^ CARIM Cardiovascular Research Institute Maastricht Maastricht University Maastricht ER The Netherlands; ^5^ Department of Oncology and Human Metabolism University of Sheffield Sheffield UK; ^6^ School of Psychology|Institute for Health Transformation Deakin University Geelong Victoria Australia; ^7^ The Australian Centre for Behavioural Research in Diabetes, Diabetes Victoria Carlton Victoria Australia; ^8^ Diabetes & Obesity Medical Unit Eli Lilly & Company Neuilly sur seine France; ^9^ Department of Psychology University of Southern Denmark Odense Denmark; ^10^ Steno Diabetes Center Odense Odense Denmark; ^11^ Department of Health and Caring Sciences Western Norway University of Applied Sciences Bergen Norway; ^12^ Novo Nordisk A/S, Medical & Science, Diabetes, Clinical Development & Project Leadership Søborg Denmark; ^13^ Eli Lilly and Company, Cardiometabolic Health Bracknell UK; ^14^ School of Medicine University of Dundee Dundee Scotland, UK; ^15^ Diabetes Advocate London UK

**Keywords:** health‐related quality of life, hypoglycaemia, person‐reported outcomes, qualitative research, quality of life diabetes

## Abstract

**Aims:**

To develop a comprehensive, in‐depth understanding of the impacts of hypoglycaemia on the quality of life of adults living with type 1 (T1D) or type 2 diabetes (T2D).

**Methods:**

Thirty‐one adults with T1D or T2D who experienced hypoglycaemia participated in semi‐structured interviews. Participants were purposively sampled by age, sex and type/duration of diabetes. Informed by a health‐related quality of life (HRQoL) framework and literature review, an interview guide explored hypoglycaemia‐related impacts on quality of life. Interviews were audio‐recorded, transcribed verbatim and analysed using Framework Analysis.

**Results:**

Impacts were coded within three overall themes (physical, psychological and social) comprising 38 subthemes. Of the 11 physical subthemes, the most discussed were sleep, physiological symptoms, leisure and exercise, eating and drinking. Of the 18 psychological subthemes, the most discussed included: awareness; cognitive burden and planning; self management and coping; worry and anxiety; autonomy, independence and control. Of the nine social subthemes, the most discussed were impacts on others and relationship with partner.

**Conclusions:**

A novel HRQoL framework highlights a comprehensive range of impacts of hypoglycaemia on physical, psychological and social functioning of people living with diabetes. These findings offer insights for clinicians, researchers and other interested parties seeking to benefit person‐centred outcomes, such as quality of life.

**What's new?**

- Hypoglycaemia impacts the lives of people living with diabetes, but there are no robust qualitative interview studies detailing in‐depth and holistically the quality of life impacts of hypoglycaemia on people living with type 1 and type 2 diabetes.
- This study generates evidence for a novel and comprehensive 38‐item conceptual quality of life framework for understanding the person‐centred impacts of hypoglycaemia, including physical, psychological and social aspects.
- Novel quality of life framework can be used to inform and benefit person‐centred outcomes for hypoglycaemia in diabetes clinical care and research, facilitating the identification, targeting and measurement of relevant quality of life outcomes.

## 1 INTRODUCTION

Hypoglycaemia is a common experience among people living with diabetes. Prevalence estimates range from 0.07% to 73.0%^1^, ^2^ and are higher in those on insulin.^3^ Hypoglycaemia episodes range from ‘self‐treated’ to ‘severe’ where, due to cognitive impairment, the assistance of another person is required for recovery.^4^ The multi‐faceted impacts of hypoglycaemia on people's daily lives include disruptions to their social, working, leisure and everyday activities^5^; difficulties with self management of symptoms^5^, ^6^; challenges to emotional and psychological well‐being^7^; interruptions to activities of daily living, such as sleep^8^ or driving^9^; and strains on interpersonal relationships.^5^, ^6^ Collectively, these factors exemplify the impacts that hypoglycaemia can have on individuals' quality of life (QoL).

Broadly, QoL can be conceptualised as an individual's ‘perception of their position in life…in relation to their goals, expectations, standards and concerns’.^10^ More specifically, health‐related quality of life (HRQoL) can be operationalised as a ‘multidimensional concept that includes the physical, psychological and social functioning associated with an illness or its treatment’.^11^ Understanding the impacts of hypoglycaemia (rather than diabetes more broadly) on the QoL of people living with diabetes is integral to its accurate conceptualisation and measurement as an outcome in clinical trials and for responsive and informed clinical care.^12^

The importance of HRQoL in diabetes care is increasingly recognised.^13^ However, in relation to hypoglycaemia, existing studies typically focus on its impacts on specific aspects of QoL, such as sleep,^8^ daily activities^9^ or emotional and psychological well‐being, including fear of hypoglycaemia.^14^ HRQoL is rarely considered holistically within hypoglycaemia, involving aspects of physical, psychological and social functioning. A recent survey study involving over 1000 adults with type 1 diabetes (T1D) demonstrated associations between hypoglycaemia and impacts in several QoL domains, including leisure, keeping fit/being active, sleep, emotional health, physical health, spontaneity, independence, work/studies and dietary freedom.^15^ Another study using the novel ‘Hypo‐METRICS’ app to record daily data on hypoglycaemia and functioning reported negative associations between person‐reported hypoglycaemia and several domains relevant to QoL, including energy levels, mood, cognitive functioning, sleep quality, memory, social functioning and fear of hypoglycaemia.^16^

Qualitative investigations into the in‐depth impacts of hypoglycaemia on QoL as a holistic concept are further limited. A recent web‐based qualitative survey study explored QoL in people living with T1D in four European countries and reported impacts in eight areas (including physical health, mental health, sex life, sleep, leisure and physical activity, work and studies, social life and everyday life).^5^ However, the depth of insight was limited by the online survey methodology and a focus on the experiences of adults with T1D only.

To our knowledge, there has been no published study exploring the impact of hypoglycaemia on QoL holistically in people living with T1D and type 2 diabetes (T2D) using a comprehensive qualitative interview methodology. In‐depth qualitative insight may aid clinicians' understanding of people living with diabetes' experiences with hypoglycaemia and help to inform clinical care. The construction of a comprehensive conceptual HRQoL model based on this approach will help improve our understanding of the breadth of impact of hypoglycaemia in research and clinical settings.

As part of the international Hypoglycaemia Redefining SOLutions for better liVEs (Hypo‐RESOLVE) project,^17^ we developed a novel HRQoL measure: the Hypo‐RESOLVE QoL.^18^, ^19^ The aim of this study is to report on the development of a comprehensive, in‐depth understanding of the impacts of hypoglycaemia on the QoL of adults living with T1D or T2D, which informed its design.

## 2 MATERIALS AND METHODS

### 2.1 Sampling and recruitment

Ethical approval was granted by the UK National Health Service (NHS) (reference: 20/NI/0048, date: 20 April 2020). All procedures were performed in compliance with relevant laws and institutional guidelines, and informed consent was obtained from all participants. Adults (≥18 years) living with T1D or insulin‐treated T2D with self‐reported experience of at least one episode of hypoglycaemia within the previous 12 months were eligible for inclusion. Participants were recruited from a large NHS hospital in the United Kingdom. Purposive sampling was used to ensure breadth in age, sex and type/duration of diabetes. Potential participants were identified using an existing hospital research database and sent a study pack directly by post. The pack contained an invitation letter, participant information sheet and consent form. Potential participants then returned the signed consent form to the clinical care team who scheduled the interview. Sampling continued until data saturation with an a priori stopping rule of no novel themes emerging for three interviews, in a sample of sufficient breadth.^20^

### 2.2 Data collection

Informed consent was acquired prior to data collection. Demographic information was extracted from clinical records. Semi‐structured interviews were conducted using a topic guide covering HRQoL themes potentially relevant to hypoglycaemia (Appendix S1), informed by a literature review^21^ and lived experience advisors. Data collection occurred between September 2020 and April 2021, and interviews were conducted online or by telephone due to COVID‐19. Two experienced qualitative researchers conducted the interviews. The researchers had backgrounds in QoL research but took care not to impose their experience and presuppositions on participants. Neither interviewer had any prior relationships with the participants. Audio recordings were transcribed verbatim and anonymised for analysis.

After the interview, participants were sent a questionnaire booklet by the clinical care team. The booklet contained measures assessing self reported hypoglycaemia awareness and HRQoL (as measured by the Hypoglycaemia Awareness Questionnaire Short Form (HypoA‐Q SF),^22^ the Hypoglycaemia Fear Survey II Short Form (HFS‐SF II),^23^ EQ‐5D‐5L,^24^ Gold Score,^25^ and Clarke questionnaire^26^).

### 2.3 Data analysis

Quantitative data were summarised descriptively. Qualitative transcripts were analysed using Framework Analysis^27^; a structured approach common in QoL research, unaligned with any specific philosophical, epistemological or theoretical paradigm.^28^ Data were initially coded independently by two interviewers, who analysed their own interviews and 50% of the other's. Analysis was informed deductively using an a priori framework based on the interview guide, and inductively as new themes emerged. Coding occurred iteratively alongside data collection, with meetings to refine the framework after each four transcripts. A final thematic framework was agreed following coding. Fresh copies of all transcripts were then ‘indexed’ by a third researcher, who coded the transcripts using the finalised framework. One of the interviewers dual indexed 20% of transcripts for agreement and met with the indexer several times to ensure consistency. Following indexing, coded data were exported into a framework matrix in Microsoft Excel, checked for consistency and an interpretative summary of each code produced.

Techniques to enhance trustworthiness of the research were based on accepted criteria.^29^ We aimed to ensure analytic credibility through significant researcher triangulation and discussion; transferability through detailed descriptions of the research context, sample, data and results (including extended supplementary material); dependability through the documentation of decisions throughout the analytic process (i.e. an auditable trail); and confirmability through ‘peer debriefing’ the findings with lived experience representatives as part of Hypo‐RESOLVE.^30^

## 3 RESULTS

Thirty‐one interviews were conducted (21 T1D/10 T2D). All participants were on insulin. Interviews lasted for a mean of 42 (range 20–77) min. Participant demographics are in Table 1.

**TABLE 1:** Participant demographics.

	*n* (%)
Age group	
18–30 years	9 (29%)
31–65 years	15 (48%)
66+ years	7 (23%)
Sex at birth	
Male	16 (52%)
Female	15 (48%)
Diabetes type	
Type 1 diabetes	21 (68%)
Type 2 diabetes	10 (32%)
Diabetes duration	
0–5 years	9 (29%)
6–10 years	5 (16%)
11–20 years	7 (23%)
21+ years	10 (32%)
Type of blood glucose monitoring^a^	
Self monitoring of blood glucose	17 (55%)
Flash glucose monitoring	2 (7%)
Continuous glucose monitoring	16 (52%)
Severe hypoglycaemia episode in the past year^b^	
Yes	4 (15%)
No	23 (85%)
Blood sugar readings under 3.5 mmol/L in the last month with symptoms^c^	
Never	3 (12%)
1–3 times	14 (54%)
1 time/week	2 (8%)
2–3 times/week	5 (19%)
4–5 times/week	2 (8%)
Almost daily	0 (0%)
Blood sugar readings under 3.5 mmol/L in the last month without symptoms^d^	
Never	19 (70%)
1–3 times	6 (22%)
1 time/week	1 (4%)
2–3 times/week	1 (4%)
4–5 times/week	0 (0%)
Almost daily	0 (0%)

	*M* (SD)	Range
EQ‐5D‐5L utility^e^	0.761 (0.178)	0.297–0.988
EQ‐5D VAS^e^	74.7 (14.68)	35–100
HFS‐SF II Total^f^	16.37 (8.39)	2–31
Gold score^g^	2.54 (1.17)	1–7
Clarke score^g^	1.54 (1.63)	0–5
HypoA‐Q SF^f^	10.4 (4.1)	4–19

*Note*: *N* = 31 (except where otherwise stated). Table adapted from Carlton et al. 2024.^18^

^a^ Responses are not mutually exclusive.

^b^ Based on Q4 of the Clarke Score ‘In the past year, how often have you had hypoglycemic episodes, where you were unconscious or had a seizure and needed glucagon or intravenous glucose’ (*n* = 27).

^c^ Based on Q5 of the Clarke Score ‘How often in the last month have you had readings <3.5 mmol/l with symptoms?’ (*n* = 26).

^d^ Based on Q6 of the Clarke Score ‘How often in the last month have you had readings <3.5 mmol/l without any symptoms?’ (*n* = 27).

^e^ Utilities calculated using ‘eq5dmap’ function on R (*n* = 28).

^f^ *n* = 27.

^g^ *n* = 26.

**FIGURE 1:**
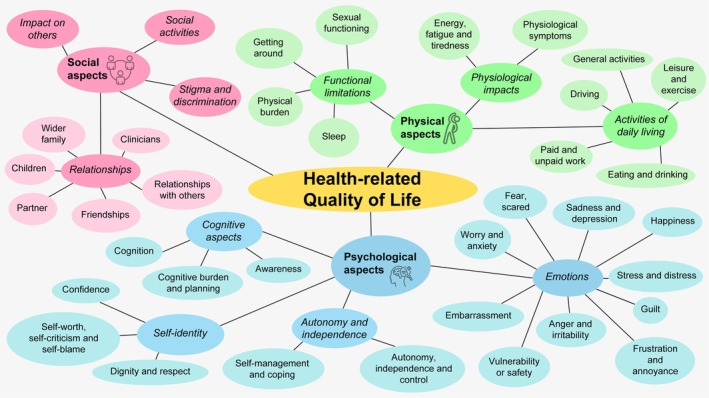
Thematic framework of hypoglycaemia‐impacted health‐related quality of life.

Data were organised into themes and subthemes (Figure 1). Data could be coded to more than one subtheme (e.g. ‘sleep’ and ‘worry and anxiety’) but are discussed below under the most suitable subtheme to avoid repetition. Supporting quotations for each theme are included in Table 2. The full thematic framework matrix with additional supporting quotes and a saturation grid showing data coverage is in Appendix S2. Eleven subthemes were coded under ‘physical aspects’, 18 under ‘psychological aspects’ and 9 under ‘social aspects’.

**TABLE 2:** Quality of life framework with example supporting quotes.

Theme	Example quotation
Physical aspects	
Activities of daily living	
Driving	‘I've probably lost confidence to drive’, cause I've not driven for 2 years and I want to drive…But like I say I don't want to go into serious hypo’ (P25)
Paid and unpaid work	‘I would never really want to do anything that was outside the sort of regular 9 to 5…I just think it would be too difficult to manage with the diabetes and hypos and things’ (P1)
Eating and drinking	‘One of the hardest things I find is, like having to eat sometimes when I really don't want to…I tried, like, jellybeans, and I tried glucose tablets, but it, I was having to physically force myself to eat’ (P9)
Leisure and exercise	‘…exercise can make it drop quite a lot…so logistically I'd probably say it would be quite hard for sport. But if you went on a run…that wouldn't be that bad because you just bring some, like an energy bar or something with you’ (P23)
General activities	‘…just kind of activities just, like, yesterday I was packing some boxes and, erm, moving some stuff. And I had a hypo then…I were [sic] proper lightheaded, and, it wasn't really on a, a time constriction, it were [sic] just, but, it was more of a, the quicker we get it done’ (P24)
Functional limitations	
Getting around	‘when it comes to travelling…I don't like to be in an environment where there's a limited supply of food, in case, I know it sounds stupid, but if there's a limited supply of food around me I just don't feel OK about it’ (P19)
Physical burden	‘I have to have a big handbag really; you can't travel light…socially going out with diabetes can be a pain because you've got to have all that…you know your hypo stop stuff in your bag as well’ (P15)
Sexual functioning	‘You have to go and do your test to make sure that you're at the right level to proceed. Erm, which can sometimes kill the mood’ (P21)
Sleep	‘I do find it hard to get a long enough quality sleep… I'm always worried about the hypo especially when I wake up in the morning and I've got a headache’ (P30)
Physiological impacts	
Physiological symptoms	‘I'll feel a bit lightheaded and then my legs will also feel a little bit wobbly…Yeah, overpowering hunger and sort of slight faintness I'd say’ (P18)
Energy, fatigue and tiredness	‘They're horrible, they really are, and they leave you feeling physically drained as well as sort of mentally, err, a little bit battered’ (P6)
Psychological aspects	
Autonomy and independence	
Autonomy, independence and control	‘I can't really go fishing on me [sic] own. Somebody needs to be with me…I can't go for a walk on my own…you can't seem to do things on your own at the moment’ (P7)
Self management and coping	‘Emotionally, if, if I'm exercising, like I say, I've, I've mentally prepared myself for it…so if I know I'm going for a run, it doesn't, if I'd end up having a hypo, it doesn't emotionally affect me, because, I try and think of the positives’ (P24)
Cognitive aspects	
Cognitive burden and planning	‘It is always there. No matter what I do…having to do blood sugars every time before I get in the car to drive…you don't know how long it's going to take for the meal to arrive, so you're then thinking, right, when will I do my insulin?…there's not a time of the day that it's not there in your mind’ (P9)
Awareness	‘The only time it's not always as good for me is in the night…when I wake up, and I sort of think I'm hypo, when I scan it [FreeStyle Libre], it shows on the graph that it's been for a little while’ (P8)
Cognition	‘Sometimes when, when you go low, like, you kind of lose the ability to like think straight…your mind kind of seizes in a certain way. Erm, so you just have to sit there and just wait’ (P22)
Emotions	
Anger and irritability	‘I get extremely irritated by it, and if somebody's around, I know if somebody's around and I'm hypo, you get where you'd swear black was white, wouldn't you’ (P10)
Embarrassment	‘I was in the middle of a supermarket when I had a hypo, so yeah, I was visible to lots of people…I'd just bumped into somebody that I knew who I haven't seen for a while…that was a bit embarrassing as well’ (P12)
Fear, scared	‘I…had to get back across London from to South London and obviously late at night there weren't [sic] places open and I did go hypo and it was like, that was quite scary’ (P10)
Frustration and annoyance	‘I think that frustration is, is one of those I'd recognise, particularly when I've done what I think is the right thing…and then I come to bed and find out it's 4.4 and I'll “oh for goodness sake.”’ (P14).
Guilt	‘They are at an age where they've retired, and still having to essentially look after me so, you know it's…I feel very guilty on that, that part’ (P16)
Happiness	‘I think once the hypo's resolved, like once I'm back in range I'm happy again, you know’ (P8)
Sadness and depression	‘It kind of makes you sad when you think that it's always, always there, something that can happen, but it's not really, it's not prevented me doing a great deal of specific things’ (P18)
Stress and distress	‘When I get a hypo, erm obviously like it's, there's a little bit of stress…you go from just, you know, enjoying your day and then all of a sudden…you have to deal with the, the hypo’ (P20)
Worry and anxiety	‘The worry that I might have one, I've never actually had one at night but the worry that I might have one then impacts my sleep because I'm like I want to stay awake’ (P31)
Vulnerability and safety	‘There is a little bit of fear when I'm out and about, you know, if I have a hypo then, you know, I might pass out and stuff, and people might not be aware of what's going on…high intensity exercise, I am aware that it could drop at any moment fairly quickly so that's another vulnerability aspect’ (P20)
Self‐identity	
Confidence	‘My confidence in physically doing things has been affected…intellectually, I don't have any problems with, what to do and how to get things done…Physically doing it, I do, right. That's, that's gone to rock bottom’ (P2)
Dignity and respect	‘I'm comfortable with my partner seeing me having a hypo, but I do feel it is undignified, especially like the mega one that I had the other night, when I was pouring with sweat and half blind’ (P1)
Self‐worth, self‐criticism and self‐blame	‘You kinda feel a bit stupid and bit that you're not done something right…you think’, ‘God I've lived with this all these years I should be able to get it bang on’…what have I done to cause a hypo’ (P15)
Social aspects	
Impact on others	‘I think it's that, putting on other people, I don't like. Do you know what I mean? I don't, I don't, I hate having to rely on somebody else. I'd have that as biggest thing’ (P3)
Relationships	
Friendships	‘A very dear friend…we used to go down and stay…But, we've not been able to get through to her that I have to have something, meals at a set time…I used to come back and I used to think “my God is it worth it?” You know. I used to be in a right state. But that is, and we don't go now’ (P13)
Partner	‘It impacted on my wife as well to be fair…I could see the worry and the fear in her…the panic, so she was really worried about me’ (P30)
Children	‘But having the hypo in front of my son or seeing him seeing me wobbly…the concern in his face that his mum wasn't very well…I don't want that anxiety on my kids’ (P26)
Wider family	‘They do worry about me and sometimes I feel like they, it's almost like I've been put on a pedestal now…and I've tried to play it down as much as possible because I don't want them to worry about me’ (P20)
Relationships with others	‘He [a co‐worker] was always quite aware and he was always looking out and making sure, making sure that I was not skipping food and things like that’ (P2)
Clinicians	‘I can remember years back, one of the consultants, he said, he looked at my HbA_1c_ and said something like “you should be ashamed of it” and I just wrung him by the neck, literally…you can get a great HbA_1c_ but actually you could be hypo all night long and fairly high during the day. But, it's all about HbA_1c_, HbA_1c_, HbA_1c_, you know? There is more to life than an [sic] HbA_1c_’ (P11)
Social activities	‘They [friends] kinda wanna like, carry on somewhere else, and it's like, you're kinda thinking in the back of your head, like, oh I don't have, in case I have an [sic] hypo, I don't have enough stuff with me’ (P21)
Stigma and discrimination	‘Yeah some people can just assume you are drunk and it, it, and they'll just like “oh go and sit down or just get out of my face kind of thing”, thinking that you're drunk or you've had one to many and it's like no I need help kind of thing’ (P31)

Abbreviation: P, participant number.

### 3.1 Physical aspects

#### 3.1.1 Activities of daily living

Activities of daily living were impacted by hypoglycaemia. Driving was less spontaneous, involved pre‐planning and could be delayed, with associated consequences (e.g. missed commitments). Some participants expressed a fear of hypoglycaemia while driving or no longer felt able to drive.

Participants perceived work performance to be impacted due to having to take time out to prevent, treat or recover following hypoglycaemia. Many participants found their employer accommodating and understanding, although some felt that they were treated differently. Hypoglycaemia, for others, limited career choices, typically to working regular and predictable hours.

Eating and drinking were frequently discussed. Participants described the need to plan their meals, know what they were going to eat in advance and eat at regular times. This limited their flexibility and made eating outside the home more difficult. Participants spoke about having to eat things or eat at times they did not want to when managing hypoglycaemia.

Many participants linked hypoglycaemia to exercise or extended physical activity (e.g. gardening). Some people avoided these activities, whereas others continued to do them with extra planning. Some people expressed fear of hypoglycaemia when exercising, with some deliberately aiming for high glucose levels to minimise the risk of hypoglycaemia. Participants differed in the extent they thought other general activities (e.g. housework, shopping) were affected by hypoglycaemia, with some doing less or making extra preparations and taking breaks.

#### 3.1.2 Functional limitations

Hypoglycaemia had a potential acute, but no lasting, effect on mobility (i.e., physically getting around). Hypoglycaemia could delay or interrupt trips and the potential for hypoglycaemia could lead to concerns about travelling (e.g., on transport or to particular places).

The physical burden of hypoglycaemia included having to carry kit around to check, prevent, manage and/or treat hypoglycaemia. Additionally, repeat finger pricking when checking blood glucose levels was linked to soreness.

Impacts on sexual functioning varied and included the need for increased planning and reduced spontaneity, which could ‘kill the mood’. A few people shared experiences of hypoglycaemia delaying or interrupting sex.

Most participants said they slept well most of the time. However, hypoglycaemia could interrupt sleep, which was associated with tiredness and impaired functioning the next day. Participants described how they could be unaware of experiencing hypoglycaemia while asleep, which could impact on quality of life via heightened worry and anxiety.

#### 3.1.3 Physiological impacts

Participants described a range of physiological symptoms during hypoglycaemia, including but not limited to sweating, hunger, feeling faint, feeling lightheaded, headache, tingling and aches, physical weakness, feeling short of breath, blurred vision, and feeling out‐of‐body. Overall, it was described as an ‘awful’ or ‘horrible’ experience, with a few people describing it feeling like ‘dying’.

Most, but not all, participants noted effects of hypoglycaemia on their energy levels, both during an episode and for a variable time afterwards, depending on severity and time of occurrence.

### 3.2 Psychological aspects

#### 3.2.1 Autonomy and independence

Some participants felt independent from other people, but not necessarily independent from things needed to manage hypoglycaemia (e.g. carbohydrates). Others felt that hypoglycaemia limited their independence from others. Perceived personal control over glucose management was important to participants, with a few describing how hypoglycaemia could leave them feeling powerless.

Most participants were self‐sufficient in managing hypoglycaemia and only a few qualitatively described needing assistance from another person. Many described the benefits of glycaemic technology in managing hypoglycaemia (e.g. through automated glucose readings). Participants described coping with hypoglycaemia as involving establishing a routine, managing food and exercise, mental preparation and acceptance.

#### 3.2.2 Cognitive aspects

Participants described how managing hypoglycaemia was always in the back of their mind like a ‘constant noise’. The need to plan was ‘24/7’ and could be ‘a grind’. People described a lack of spontaneity and flexibility. Some participants described pushing back against this and actively taking risks. For some, checking blood glucose regularly provided comfort.

Awareness or the ability to detect ‘going hypo’ differed across people, time and context. Most participants had awareness of the onset of hypoglycaemia. However, some noted that their warning signs had changed over time, and others had experienced hypoglycaemia without awareness. Awareness was impeded during sleep or when concentrating on other things. This could impact quality of life through heightened worry and anxiety or the avoidance of activities.

Individuals' cognitive capacity (ability to think, concentrate and function mentally) was impacted acutely during hypoglycaemia and potentially for a short time after. Participants discussed being unable to think clearly, getting confused and isolated impacts on memory. Participants described this as ‘brain fog’.

#### 3.2.3 Emotions

Hypoglycaemia could cause people to become irritable, short tempered and argumentative. This was a common experience for many, but not all, participants who recalled instances of getting impatient and angry with others during hypoglycaemia.

Experiencing hypoglycaemia in public, or around certain people, was a cause of potential embarrassment for some, but not all. This was due largely to being perceived by others as ‘odd’ or ‘out of control’.

Fear was associated with potentially experiencing hypoglycaemia and hypoglycaemia itself being frightening, particularly when not resolving promptly, when treatment was not available, or when it became severe. Fear of hypoglycaemia was particularly prominent in certain situations, such as when driving, looking after children or when asleep. A few people did not report fear related to hypoglycaemia and others stated their fear had dissipated over time.

Frustration was a common experience, associated with the relentless management and treatment of hypoglycaemia, as well as having episodes and the resultant disruption. A prevalent cause of frustration was experiencing hypoglycaemia without a clear explanation or when participants perceived they had otherwise ‘done everything right’.

While some participants did not experience guilt, others shared how hypoglycaemia could make them feel guilty by impacting others. Examples included the perception that hypoglycaemia may have ruined an outdoor activity, that participants were unable to contribute to childcare and related responsibilities, or felt responsible for other people's worries.

While happiness was not discussed by many, a few people discussed how they felt happy if their glucose management was going well or after hypoglycaemia resolved. Conversely, hypoglycaemia was associated with instances of low mood, sadness and depressive mood but not necessarily mental health problems. Participants shared how not being able to ‘control’ their glucose levels, or finding it difficult to treat hypoglycaemia, could lead to them feeling down. Furthermore, the experience of hypoglycaemia could leave people feeling more emotional and/or lead to low mood. However, this was not universal.

Stress was a term used by some people to describe instances related to hypoglycaemia, such as when they could not explain why they experienced hypoglycaemia or during the episode itself and for a while afterwards. Hypoglycaemia itself, particularly when severe, was associated with a heightened state of stress and distress in some cases (e.g. a ‘fight or flight’ response).

Several participants did not experience any anxiety or worry and would simply deal with hypoglycaemia as it happened. For others, worry and anxiety was related to the potential for hypoglycaemia when unprepared, in unfamiliar places, or without someone around. Anxiety and worry could also present during hypoglycaemia (e.g. if a glucose reading was particularly low or unresponsive to treatment). Some people shared that they were worried about hypoglycaemia while asleep, or anxious about the potential for repeated episodes of hypoglycaemia when one had occurred earlier in the day.

People expressed concerns about vulnerability and safety. These were particularly pronounced when there was a risk of developing hypoglycaemia with no‐one around, in remote places or without treatment available. This included concerns when sleeping alone or experiencing hypoglycaemia in front of others who were not necessarily perceived as competent in dealing with it. Participants also expressed concern for the safety of others in their care (e.g. children) if they had a severe event.

#### 3.2.4 Self‐identity

For some people, hypoglycaemia affected their confidence to go and do ‘normal things’, including physical tasks such as wallpapering or driving. Some participants described how glycaemic technologies (e.g. automated glucose monitoring) gave them more confidence to do tasks.

Most, who commented on the issue, felt like they were generally treated with dignity and respect. Though one participant recalled being treated with a lack of respect in their workplace. One participant felt they gave away part of their ‘privacy’ by having to warn people about hypoglycaemia. Another described the experience of hypoglycaemia as ‘undignified’.

While not universal, most people who commented on the issue identified instances of self‐criticism and self‐blame, particularly if they perceived that they had done something to cause hypoglycaemia. Some participants were their own biggest critic and reported feeling ‘stupid’ or like a ‘failure’ that they let hypoglycaemia happen. Self‐criticism was linked to impacts on self‐worth.

### 3.3 Social aspects

#### 3.3.1 Impact on others

One of the most discussed topics was the perceived impact on others. While not all participants felt this way, most identified a negative influence of hypoglycaemia on other people. This included having to rely on others more, inconveniencing others, increased irritability and/or causing others to worry or feel scared.

#### 3.3.2 Relationships

Many of the social impacts of hypoglycaemia were discussed in the context of various relationships, with friends, partners, children, the wider family, clinicians and others. Most participants reported minimal or no impacts on the types or quality of friendships kept, with many reported as good and supportive. The largest impact on friendships was through restricting or altering activities, such as no longer going to visit and stay with friends due to irregularity in the times of meals provided.

For most people, the relationship with their partner was unaffected. They described their partner as accepting and responsive to their needs regarding hypoglycaemia. However, for some, hypoglycaemia was associated with arguments with their partner and/or instances of perceived stress and worry. Concern over the impact of hypoglycaemia extended to other members of the family, including parents, children and grandchildren. Some described how they were embarrassed or uncomfortable with their wider family seeing them during hypoglycaemia or when managing their glucose and would try to avoid this. Others discussed the potential for distress if children witnessed hypos and potential interference with parental caring responsibilities.

Beyond one's partner, family and friends, participants described mixed experiences with others. Some participants described how the experience of hypoglycaemia could be alienating as other people did not understand what they were going through or how to manage it. Others described instances of support and understanding from others (e.g. co‐workers).

Finally, mixed experiences with healthcare professionals were shared. Some reported positive experiences, whereas some described negative instances where clinicians had been patronising or condescending about their glucose management.

#### 3.3.3 Social activities

For most people, hypoglycaemia did not stop them doing things socially but meant that activities required more planning and adaptation. Participants described being more cautious and/or avoiding certain elements of activities, such as social drinking because of perceived impacts on glucose levels and interactions with insulin. Overall, hypoglycaemia impeded spontaneity in socialising.

#### 3.3.4 Stigma and discrimination

Some participants did not perceive any discrimination related to hypoglycaemia. However, others reported perceived instances of people giving them ‘funny looks’ when checking their glucose levels or drinking their own (sugary) drinks in a restaurant. Some people explained that they felt conscious about others looking at them or thinking badly of them if they were to experience hypoglycaemia in public. One participant described being treated differently at work when their colleagues knew about their hypoglycaemia (e.g. given roles with less responsibility).

## 4 DISCUSSION

While previous research has attested to the impacts of hypoglycaemia on QoL, this study makes a unique contribution as the first to explore in‐depth HRQoL impacts through interviews with adults living with T1D and T2D. Our research demonstrates wide‐ranging impacts of hypoglycaemia on physical, psychological and social aspects of people's lives. The findings are largely consistent with a prior qualitative survey study exploring QoL impacts of hypoglycaemia in T1D, with impacts recorded in domains such as relationships and social life, work and studies, leisure and physical activity, everyday life, sleep, sex life and mental health.^5^ However, additional in‐depth insights are afforded into these and broader themes in this paper, such as an expanded understanding of the emotional impacts of hypoglycaemia; issues of autonomy and independence, vulnerability and safety; stigma and dignity; and pervasive cognitive burden.

Acknowledging that coding coverage is likely to be a crude estimate of relative importance, the subthemes most discussed included eating and drinking; leisure and exercise; paid and unpaid work; energy, fatigue and tiredness; physiological symptoms; sleep; autonomy, independence and control; awareness; cognitive burden, planning and obsessive behaviour; worry and anxiety; self management and coping; and impact on others. Similar areas were noted as important to QoL for adults with T1D in the abovementioned qualitative study.^5^ Where aspects of QoL relevant to hypoglycaemia have been valued societally by members of the public, not being able to do what you want to do in life, feeling exhausted, having to take time out during work activities and being an inconvenience to others were perceived as having the biggest perceived reduction in QoL.^31^

The links between daily activities—including exercise, employment and sleep—and hypoglycaemia have been well documented.^32^ The association between hypoglycaemia and impaired sleep, for example, has been demonstrated in numerous studies.^8^, ^15^, ^33^ This includes impacts on both sleep duration and sleep quality.^33^ The psychological impacts of hypoglycaemia, including independence, cognitive burden and anxiety have been noted elsewhere.^5^, ^15^ Chatwin and colleagues described how adults with T1D were ‘always “working on” hyoglycemia’.^5^ Our research provides deeper insights and supporting evidence for this pervasive cognitive burden experienced by people living with T1D or T2D. Finally, the social consequences of hypoglycaemia (i.e. impact on others) have, perhaps, been less well studied than the physical and psychological outcomes, yet supporting evidence exists.^5^, ^34^ In particular, relationships and social life have been identified as the most important domain of QoL related to hypoglycaemia by people living with T1D,^5^ suggesting that this may be a key outcome for QoL interventions. There have also been recent efforts to combat social stigma and discrimination in diabetes,^35^ which is an important consideration relevant to hypoglycaemia.^36^

Potential limitations of this study include that all participants were recruited from a single, albeit large, UK NHS hospital. It is possible that geographical variation in HRQoL impacts exists that was not captured in this study. Second, data were collected during the COVID‐19 pandemic, which may have affected responses, such as the focus, contextualisation or extent of HRQoL impacts. Third, the relative importance of the 38 HRQoL impacts identified was not explored in this study, although coding coverage and data saturation is provided. Fourth, this qualitative study focused on common potential HRQOL impacts across individuals and was neither designed nor sufficiently powered to enable between‐group comparisons (e.g. based on diabetes type). Instead, this issue is addressed quantitatively elsewhere.^18^

In conclusion, a 38‐item framework has been produced that comprehensively charts the impacts of hypoglycaemia on HRQoL for adults living with T1D or T2D, informed by rich qualitative data. This framework has been used to inform the development of a novel measure of HRQoL specific to the impacts of hypoglycaemia.^18^, ^19^, ^31^ It has additional value in illuminating the lived experience impacts of adults who experience hypoglycaemia and informing potential person‐centred outcomes relevant to hypoglycaemia in research, clinical and social settings.

## AUTHOR CONTRIBUTIONS

**Philip A. Powell:** Study conceptualisation, data curation, formal analysis, investigation, methodology, project administration, resources, supervision, visualisation, writing—original draft, writing—revisions. **Sé M. Frances:** Data curation, formal analysis, visualisation, writing—original draft, writing—revisions. **Bastiaan E. de Galan:** Study conceptualisation, funding acquisition, supervision, writing—revisions. **Simon Heller:** Study conceptualisation, funding acquisition, supervision, writing—revisions. **Jane Speight:** Study conceptualisation, funding acquisition, methodology, supervision, writing—revisions. **Myriam Rosilio:** Study conceptualisation, funding acquisition, writing—revisions. **Frans Pouwer:** Study conceptualisation, funding acquisition, methodology, writing—revisions. **Mari‐Anne Gall:** Study conceptualisation, writing—revisions. **Christopher J. Child:** Study conceptualisation, writing—revisions. **Rory J. McCrimmon:** Study conceptualisation, funding acquisition, writing—revisions. **Ken Tait:** Study conceptualisation, writing—revisions. **Jill Carlton:** Study conceptualisation, data curation, formal analysis, funding acquisition, investigation, methodology, project administration, resources, supervision, visualisation, writing—original draft, writing—revisions.

## FUNDING INFORMATION

This Hypo‐RESOLVE project received funding from the Innovative Medicines Initiative 2 Joint Undertaking (JU) under grant agreement no. 777460. The JU receives support from the European Union's Horizon 2020 research and innovation programme and EFPIA and T1DExchange, JDRF, IDF, HCT. The authors are solely responsible for the content of this work, which reflects only the authors' views, and the JU is not responsible for any use that may be made of the information it contains. JS is supported by core funding to the Australian Centre for Behavioural Research in Diabetes, derived from the collaboration between Diabetes Victoria and Deakin University. The funders had no role in the study design; collection, analysis and interpretation of data; in writing the report or decision to submit the article for publication.

## CONFLICT OF INTEREST STATEMENT

SH has received consulting fees from Eli Lilly (payment to International Hypoglycaemia Study Group), consulting fees from Zealand Pharma (payment to Institution), consulting fees from Zucara Pharma (payment to Institution), consulting fees from Novo Nordisk as part of Member of International Review Board (payment to Institution) and payment for expert testimony from patent case (personal payment); has participated on a Data Safety Monitoring Board or Advisory Board at Eli Lilly (payment to Institution) and is Chair of International Hypoglycaemia Study Group. JS has served on advisory boards for Janssen, Medtronic, Omnipod, Roche Diabetes Care, Sanofi Diabetes and Vertex; received unrestricted educational grants and in‐kind support from Abbott Diabetes Care, AstraZeneca, Medtronic, Roche Diabetes Care and Sanofi Diabetes; received sponsorship to attend educational meetings from Medtronic, Roche Diabetes Care and Sanofi Diabetes and consultancy income or speaker fees from Abbott Diabetes Care, AstraZeneca, Insulet, Medtronic, Novo Nordisk, Roche Diabetes Care, Sanofi Diabetes and Vertex. In all cases, JS's research group (ACBRD) has been the beneficiary. MR has stock or stock options at, and is an employee of, Eli Lilly and Company. M‐AG is a shareholder and employee of Novo Nordisk A/S. CJC is a shareholder and employee of Eli Lilly and Company. RJM has received honoraria for lectures, presentations, speakers bureaus or educational events from Sanofi. JC is a member of Diabetes UK Research Steering Group (RSG). PAP, SMF, BEdG and FP have no conflicts of interest to declare.

## Supporting information


**Appendix S1.** Semi‐structured interview topic guide.

**Appendix S2.** Thematic framework matrix.
