# Supplementary material for: ‘There is more to life than HbA1c ’: A comprehensive qualitative framework from the Hypo‐RESOLVE project evidencing the impacts of hypoglycaemia on the quality of life of adults living with type 1 or type 2 diabetes
Source: Diabet Med. 2026 Feb 9;43(5):e70240. doi: 10.1111/dme.70240 (PMC13074142; doi:10.1111/dme.70240)
Supplement: Supplementary file 1 — Appendix S1. Semi‐structured interview topic guide. [file DME-43-e70240-s002.docx]

**UNDERSTANDING THE IMPACT HYPOGLYCAEMIA HAS ON PEOPLE LIVING WITH DIABETES**

**Interview Schedule**

**Before the interview:**

- The interviewer will introduce themselves, and thank you for agreeing to take part.
- They will provide some background to this research:

*“The overall purpose of this project (called the Hypo-RESOLVE project) is to reduce the burden and consequences of hypoglycaemia for people living with diabetes. As part of the Hypo-RESOLVE project, we want to understand how hypoglycaemia can affect the day-to-day lives of people living with diabetes.*

*This research is funded by Innovative Medicines Initiative (IMI), a joint undertaking of the European Commission and the European Federation of Pharmaceutical Industries and Associations (EFPIA), T1D Exchange, JDRF, International Diabetes Federation (IDF), and the Leona M. and Harry B. Helmsley Charitable Trust.*

*The aim of the study is to explore what impact hypoglycaemia has on quality of life (QoL) in people living with diabetes. By understanding how and in which ways hypoglycaemia affects people’s QoL, people who make health care decisions, including doctors, will know what to measure when they are testing any new treatments designed to reduce the impact of hypoglycaemia and improve QoL. While we may touch on things that may have affected you in the past, or may affect you in the future, we are interested in the things that are important to you today, in the present.”*

- The interviewer will go through some important points with you:
  - With your permission we would like to record this interview. This will help us to focus on the things you say.
  - Everything you say is confidential. Information you share in this interview will not be passed on to others outside of the research team, except if it is information that suggests you may be a threat to yourself or others, which we have a duty to report. Any quotes used in reports will be anonymised.
  - In the interview, we might discuss things you may find difficult. You do not have to answer any questions you do not want to. If there are any questions that you are uncomfortable with, or you find difficult, just let us know and we can move on.
  - The interview will take us approximately an hour to complete. However, you can stop the interview at any time, or take a break, if you wish to do so.
- The interviewer will provide a further copy of the information sheet, and ask you to complete a consent form. After the interview, you will be asked to fill in some questionnaires to find out a bit more about your quality of life.
- The interviewer will ask you if there is anything you would like to ask us before we begin.

**Questions to cover during the interview:**

1. I’d like to start by asking what you understand by the term ‘quality of life’. What does it mean to you?
2. What aspects of your life are important for your quality of life?
3. So, you’ve mentioned X, Y, Z. Can we talk about each of these in turn with you telling me how hypoglycaemia affect each of these? *[Then discuss each in turn…]*
4. So, let’s discuss a few things that you didn’t mention spontaneously… I’d like to start by asking you about several areas of your life in turn, to see in each area which aspects are important to you personally. Let’s start by talking about the physical factors that may affect your quality of life. What impacts on your physical functioning does hypoglycaemia have?

[*prompts: activities of daily living (include self-care, work, leisure activities, driving, etc), energy (including fatigue), mobility, sexual functioning, sleep*]

1. Thank you. Let’s move on now to talk about your psychological health. In what ways does hypoglycaemia affect way you feel emotionally and/or psychologically?

[*prompts: does it affect your happiness or mood?; do you feel depressed?; do you feel angry or irritable?; do you feel anxious?; are you able to cope with living with diabetes and hypoglycaemia?, what coping strategies do you have? Do you have any worries or fears?; do you feel stressed or in distress?; do you feel like a burden?; do you have concerns over your own safety (or the safety of others around you)?*]

1. Thanks. Let’s move on to talk about the social aspects of your life. In what ways does hypoglycaemia affect your ability to socialise and take part in things with others?

[*prompts: participation in social and leisure activities; friends and social networks; intimate relationships with others; effects on studying or work*]

1. Okay, thank you. Can we talk now about your sense of identity and independence? What effects do you think hypoglycaemia has on your independence and the way you are treated by others?

[*prompts: dependence on others and self-care; autonomy; dignity and respect from others; fatigue*]

1. Thank you very much. Aside from the topics we have covered so far, are there any other ways that hypoglycaemia affects your quality of life? This could be anything that is important to you. Please share as much information as you are able.

**Close:**

The interviewer will turn off the recording and ask you if you have any questions about the project. They will then thank you for your time.
